# A Linear Empirical Model of Self-Regulation on Flourishing, Health, Procrastination, and Achievement, Among University Students

**DOI:** 10.3389/fpsyg.2018.00536

**Published:** 2018-04-13

**Authors:** Angélica Garzón-Umerenkova, Jesús de la Fuente, Jorge Amate, Paola V. Paoloni, Salvatore Fadda, Javier Fiz Pérez

**Affiliations:** ^1^School of Psychology, Fundación Universitaria Konrad Lorenz, Bogotá, Colombia; ^2^Department of Psychology, University of Almería, Almería, Spain; ^3^Universidad Autónoma of Chile, Santiago, Chile; ^4^National Research Council, Universidad Nacional Río Cuarto, Río Cuarto, Argentina; ^5^Prevention Service, Università degli Studi of Sassari, Cedenia, Italy; ^6^Department of Psychology, Università degli Studi Europea di Roma, Rome, Italy

**Keywords:** self-regulation, procrastination, flourishing, health, academic performance, university students

## Abstract

This research aimed to analyze the linear bivariate correlation and structural relations between self-regulation -as a central construct-, with flow, health, procrastination and academic performance, in an academic context. A total of 363 college students took part, 101 men (27.8%) and 262 women (72.2%). Participants had an average age of 22 years and were between the first and fifth year of studies. They were from five different programs and two universities in Bogotá city (Colombia). A validated *ad hoc* questionnaire of physical and psychological health was applied along with a battery of tests to measure self-regulation, procrastination, and flourishing. To establish an association relationship, Pearson bivariate correlations were performed using SPSS software (v. 22.0), and structural relationship predictive analysis was performed using an SEM on AMOS software (v. 22.0). Regarding this linear association, it was established that (1) self-regulation has a significant positive association on flourishing and overall health, and a negative effect on procrastination. Regarding the structural relation, it confirmed that (2) self-regulation is a direct and positive predictor of flourishing and health; (3) self-regulation predicts procrastination directly and negatively, and academic performance indirectly and positively; and (4) age and gender have a prediction effect on the analyzed variables. Implications, limitations and future research scope are discussed.

## Introduction

To this day, the *self-regulation* (SR) construct has shown a great explanatory capacity on the behaviors involved with health and education (Mann et al., 2013; Zimmerman and Kitsantas, 2014; Panadero, 2017). Also, previous and recent studies have informed of evidence in relation to the behavioral consequences of task postponement -*procrastination*- as a negative or avoidance conduct in the academic environment (Clariana, 2013; Sommer, 2013; Balkis and Duru, 2017), and of the positive experience -flourishing- on health (Pattinson and Edgar, 2016). However, despite the previous evidence, the relation between these three psychological constructs has not been established with high precision.

### Self-regulation: the theory of self- vs. externally-regulated learning™

Brown (1998) conceptualized *self-regulation* (SR) from the tendency of individuals regarding their particular ability to plan and flexibly manage their behavior. SR has received particular attention in recent years as an essential factor to better comprehend health and disease, as a result of healthy habits and individuals' capacity to set and maintain healthy goals (de Ridder and Kuijer, 2006; Mann et al., 2013). In fact, SR has been considered, based on recent evidence, as a variable or construct of the meta-behavioral order, meaning, a meta-skill or skill to manage the cognitive, affective and motivational abilities. These are shown in the model *Competence of Learning, Studying and Performing under Stress*, with the acronym CLSPS™ (de la Fuente, 2015; de la Fuente et al., 2017a).

This self-regulation vs. externally-regulated learning (SRL vs. ERL) theoretical approach (de la Fuente, 2015, 2017) predicts that the individuals' may be characterized on a behavioral continuum: *Self-regulatory, A-regulatory, or De-regulatory behavior*. There is evidence that people may have different degrees of personal self-regulation (high-medium-low), alluding to the extent and to the number of practices they employ to exercise their behavioral regulation (de la Fuente et al., 2015). *Self-regulation* (SR), *or high in self-regulation*, may be considered as the degree of a person's *positive proactivity* in its active and adequate management of the regulation of well-being and health (Brown, 1998). *A-regulation* (AR), *or medium in self-regulation*, may be defined conceptually as the lack of proactivity and so equivalent to the concept of behavior *reactivity* (Zimmerman and Labuhn, 2012). *De-regulation* (DR), *or low in self-regulation*, may be defined as the degree of *negative proactivity*, that is, of active and inadequate management to regulate one's behavior. This de-regulation avoids the effort involved in proactive self-regulation of health, and of the procrastination (Clariana, 2013; Balkis and Duru, 2017). In a previous research report, examples of this theoretical formulation were presented (de la Fuente, 2017, pp. 3–4, See Table 1).

**Table 1:** Conceptual continuum and typologies of each self-regulatory behavior (reproduced with permission).

**Characteristics of the person**	**Self-regulation (SR) High- Moderate- Low POSITIVE PRO-ACTIVITY (+1)**	**A-regulation (AR) No regulation RE-ACTIVITY (0)**	**De-regulation (DR) Low- Moderate- High NEGATIVE PRO-ACTIVITY (−1)**
	*Before* Self-analysis of tasks Self-defines goals Self-motivation	*Before* No analysis of tasks No goals No motivation	*Before* Erroneous self-analysis Erroneous goals Self-demotivation
	*During* Self-observation Self-analysis Self-correction	*During* No self-observation No supervision No self-correction	*During* Self-distraction Cognitive self-avoidance Self-impediment Procrastination^*^
	*After* Self-reflection Self-attributions Positive self-affects^*^	*After* No reflection No attributions No affects	*After* Erroneous self-assessment Erroneous self-attributions Negative self-affect
**Type of activity**	**Self-regulatory (SR) High-Moderate-Low PRO-ACTIVITY (**+**)**	**A-regulatory (AR) No regulation RE-ACTIVITY (** = **)**	**De-regulatory (DR) Low-Moderate- High PRO-ACTIVITY (**−**)**
Academic	Self-regulated learning	No norms/limits	Self-induction impediment
Road safety	Self-regulation in driving	No norms/limits	Self-induction of risks
Health	SR in Health	No norms/limits	Self-induction of excesses
TV	SR in TV	No norms/limits	Self-induction of excesses
Family	SR in family	No norms/limits	Self-induction of risks
Technology of Information and Communication (TIC)	SR in TIC	No norms/limits	Self-induction of excesses
Sexual	SR in risky sexual behavior	No regulation	Self-induction of risks
Violence	SR in harmonious relations	No norms/limits	Self-induction of excesses
Spouse/partner	SR in interaction	No regulation	Self-induction of excesses

* *Place of positive self-affect (flourishing) and procrastination in this theoretical model*.

### Self-regulation, flourishing, and health

The hypothesis arises, from *SRL* vs. *ERL* theory (de la Fuente, 2015, 2017), that a higher SR level has associated with it a high degree of *well-being* and *health* since SR could be a meta-skill that would contribute actively to those. Thereby, flourishing and physical and psychological health would be correlated with a higher level of self-regulation. Seligman (2011) defines flourishing as a set of characteristics such as positive emotions, enthusiasm and a sense of purpose—a concept related to optimism, resilience, vitality, self-determination and positive interpersonal relations. Therefore, *flourishing* is the combination of psychological and subjective well-being associated with a meaning and purpose in life (Pozo et al., 2016).

For the mental health study, flourishing would be a positive form of mental health, beyond the sole absence of disease (Keyes, 2007). Although flourishing has been so far better studied in health, its role in educational contexts has also started to be studied. Although some evidence indicates that flourishing predicts the academic grades, both self-informed and objective—once demographic and other variables of subjective well-being are controlled—there is not enough research establishing a relationship between academic performance and flourishing (Datu, 2018). Moreover, flourishing has been explicitly studied in academic contexts concerning the student's well-being, academic achievements and other variables such as stress or coping strategies. As an example, some reports of college students indicate that flourishing is predicted by coping skills. At the same time, coping skills and flourishing are predicted by the mindfulness (Akin and Akin, 2015).

Some previous studies have investigated the relationship between SR, procrastination and positive psychology measures such as well-being or flow, through structural equations model (SEM). Balkis (2013) found that rational beliefs about studying mediate the relation between procrastination, life satisfaction, and academic performance in the same way as life satisfaction mediates the relationship between procrastination and the rational beliefs about studying. Apparently, rational beliefs mediate the emotions and functional and dysfunctional behaviors alike. Likewise, there is evidence that self-efficacy has a role as a predictor of flow and procrastination in academic performance (Vinothkumar et al., 2016) and the strategies of motivational and educational regulation, academic performance and procrastination are related to it. This evidence indicates that higher the use of management strategies leads to less procrastination and better performance (Grunschel et al., 2016). The use of emotional and academic regulation strategies results in the affective and cognitive well-being of the students; on the contrary, the negative affective well-being and low academic performance are related to procrastination.

### Self-regulation, procrastination, and reasons to procrastinate

It has been found that procrastination, as a *deregulation behavior* (de la Fuente, 2017) is associated with a treatment delay and the existence of a lesser amount of well-being conducts (Rothblum et al., 1986; Sirois, 2007). It is also related to disease development, having as a mediator, high stress levels, and greater treatment delays (Sirois and Tosti, 2012). As an example, in a study with persons suffering from hypertension and cardiovascular disease, it was found that procrastination is more closely related to maladaptive general behaviors of the patients than with the missed mandatory health checks, being a factor of primary vulnerability in the disease management (Sirois, 2015). Likewise, there is evidence that points out the relationship between chronic procrastination and stress, which could have consequences such as mental distress (coping inability) and physical distress (Ferrari and Díaz-Morales, 2014). Kiamarsi and Abolghasemi (2014) found in students that self-efficacy and procrastination explain 40% of the psychological vulnerability variance (which included physical, anxiety and depression factors).

This study is based on a perspective of motivation and volition psychology, understanding that “procrastination is typically taken as an irrational or a self-defeating delay, to be worse off for putting off” (Steel and Klingsieck, 2015, p. 73). Consequently, a component of procrastination is “delaying,” but the delays do not necessarily end in procrastination. Steel and Klingsieck (2015) propose to better define procrastination, to take into account three elements: (1) intention, (2) voluntary delays, and (3) pathological tendency. When defining procrastination as the voluntary delay in the course of an action, it can be assumed that this variable is closely related to the processes of SR, to the point that it has been characterized as quintessential to self-regulation failure (Steel, 2007). There are different explanations for the gap between intentions and behavior from SR. Kroese and de Ridder (2016) point out that persons take liberties and justify their “bad” behavior, rather than a lack of skills or impulsive conducts. Therefore, persons “may deliberately set aside their health goals and prioritize satisfaction of immediate needs when they have a license to do so” (p. 315). These authors relate this intentional self-regulatory failure with procrastination, which is the way a person knowingly and willfully fail, allowing oneself not to do something. In this sense, the knowledge of the reasons to procrastinate (as a psychological mechanism), as well as the number of procrastination behaviors (that refers to the habit) is of greater relevance. Just so, is possible to understand the cognitive de-regulatory component of procrastination, as a mechanism, by the subject, of maladaptive ideations, as the cited theory establishes. In the same way, it would be understood that SR predicts procrastination, health, well-being, and academic performance variables. This concept is similar to the *proactive deregulatory behavior* (de la Fuente, 2017).

### Self-regulation, procrastination, academic performance and health

From *SRL* vs. *ERL Theory* (de la Fuente, 2015, 2017), it has been hypothesized that a greater SR level has to have associated with it a lesser procrastination level and consequently, a higher level of procrastination a lower level of performance. Regarding the relationship between procrastination and academic performance, Steel (2007) found—in a meta-analytic review—a weak but consistent negative relationship between both variables. Consistently procrastinators show negative correlations with average cumulative grades, average grades for a particular course, results of a final exam or homework grading.

Higher academic performance can be predicted by the type of goal orientation set by the students and through a high level of metacognitive self-regulation skills (Dekker et al., 2016). Kitsantas et al. (2008) found that previous academic performance together with motivation and self-regulation variables explain 47% of the variance of student grading at the end of their first college year. Zimmerman and Kitsantas (2014), through SEM, found SR as a latent factor which, unlike the “self-discipline” variable, significantly predicts the academic performance of students.

Regarding the relationship between procrastination and health, there is evidence that the improvement in time management skills, which would lead to a decrease in academic procrastination, enhances the efficient use of study time and other positive effects such as a reduction of students' anxiety and a better management of social and recreational relations (Senécal et al., 1995). As a consequence, this improvement brings a better wholesome control of life for the successful development of health and psychological well-being (Kaya et al., 2012).

However, although there is evidence that relates procrastination and health negatively, authors such as Kroese and de Ridder (2016) point out that the more significant part of this evidence comes from indirect studies where the relationship between general procrastination and health is implicit. These authors propose that procrastination should be considered directly and differentiate it from issues such as problems of inhibitory control or low self-control skills. Likewise, procrastination cannot be explained by one perspective alone: the integration of the different aspects is required to achieve its concept and dynamics (Klingsieck, 2013). In this sense, the present study analyzes, the differential effect of SR levels in relation with procrastination; and its association and effect on health and flourishing in an education context.

### The role of age and gender

Although it has not been the primary focus of self-regulation, procrastination or flourishing study, in literature, some gender and age differences are reported, which are of interest in these constructs and other related ones. It seems that there is a progressive diminishing of procrastination with age, and higher levels of procrastination in men compared to women have been reported (Carranza and Ramírez, 2013; Cardona, 2015). Likewise, Robotham (2012) points out that those who combine a day job with studying, consider it inconvenient due to the lack of time to perform their academic tasks, which could lead to procrastination behaviors.

Academic time management is a fundamental component of SR in the learning process (Pintrich et al., 1993; Barber et al., 2009). Some studies have found better time management skills in women when compared to men (Soares et al., 2011; Kaya et al., 2012; Durán-Aponte and Pujol, 2013; Pehlivan, 2013; Garzón et al., 2017b). In a meta-analysis that considered 187 investigations (Huang, 2013), gender and age differences were found in academic self-efficacy. These varied by study area and age group.

In regard to the psychological well-being differences between men and women, it was found that men have higher scores in *physical self-concept, automatic thoughts (positive), constructive thinking, cognitive flexibility, total self-concept, and fortitude* while women tend to have higher scores in *the expression of affect, somatic symptoms, and religious well-being* (Roothman et al., 2003). In Europe, scarce differences have been found between men and women concerning the level of flourishing, that would be better determined by the education level and income; although depending on the country, the level of flourishing can decrease with age (Huppert and So, 2009).

### Aims and hypotheses

The concept of s*elf-regulation behavior* proposed by the *Theory of Self- vs. Externally-Regulation Learning* (de la Fuente, 2015, 2017), has predicted and contributed evidence to understand *behavioral self-regulation* as a personal construct that can determine and predict, positively, the type of coping strategies focused on the problem, resilience level or engagement (de la Fuente et al., 2015; Artuch-Garde et al., 2017). On the other hand, the theory has evidenced that the *behavioral de-regulation* determines or predicts maladaptive behaviors such as procrastination (Garzón and de la Fuente, in review). However, these relationships have only been partially proved, which justifies the need to do it in this study, from an interdependence and structural relationship perspective.

The general aim of the study was to establish the linear association and prediction relations of *self-regulation*-as a macro-construct- on flourishing and health, on the frequency and reasons to procrastinate, and academic performance. The hypotheses are, (1) it is expected that self-regulation shows a positive linear relationship of association and prediction in regard to flourishing and health (physical and psychological) of the students; (2) it is expected to find a negative linear relationship, association and prediction, between self-regulation, in regard to procrastination (frequency and reasons), and the performance of the students; (3) finally, it is expected that *gender* and *age* predict the above relationships (Artuch-Garde et al., 2017). Based on the previous evidence, we expect that the female *gender* has a positive relationship with flourishing and health, as well as a negative relationship with procrastination; also, that an advanced *age* has a negative relationship with health and performance (Roothman et al., 2003; Huppert and So, 2009; Özer et al., 2009; D'Lima et al., 2014).

Understanding that these factors are interrelated, and are an additional approach to different psychological elements of the individuals', it is expected that they offer a more sophisticated level of explanation and a superior predictive value on procrastination, health and academic success of students. Nowadays, within the area of applied research in academic contexts, the trend is to integrate and structure different factors to constitute predictive models that increase the capacity to comprehend on which reasons the success and life quality of students rely upon (Robbins et al., 2004; Kitsantas et al., 2008; de la Fuente et al., 2015).

## Methods

### Participants

A total of 363 college students took part in the investigation: 101 men (27.8%) and 262 women (72.2%), with ages ranging from 16 to 53 years old with an average of 22, from two universities in Bogotá (Colombia). 217 participants were from Fundación Universitaria Konrad Lorenz and 146 from Universidad El Bosque. By programs, 249 students were from Psychology (68.6%), 39 from Management and Business (10.7%), 37 from Engineering (10.2%), 27 from Health-related programs (7.4%) and 11 students from other study areas (3%). 127 (35%) students were enrolled in their first year, 106 (29%) in their second year, 96 (26.4) in their third year, 24 (6.6%) in their fourth year, and 10 (2.8%) in their fifth year. 290 (80%) belonged to day programs and 73 (20%) to night programs. As for dedication, 265 (73%) were full-time students and 98 (27%) were part-time students and part-time workers or had other responsibilities.

### Instruments

#### Short self-regulation Spanish validated questionnaire, short-SR (Pichardo et al., 2014)

As a self-regulation measure, the abbreviated Spanish adaptation of the Self-Regulation Questionnaire (SRQ) was used, which in turn was created based on the original questionnaire by Brown et al. (1999), which seeks to measure general self-regulation behavior that leads people to plan and direct their own behavior in a flexible manner according to the demands of the environment (Brown, 1998). The Short-SR consists of 17 items and four dimensions (Goal setting, Perseverance, Decision-Making and Learning from mistakes), obtained through Exploratory and Confirmatory Factorial Analysis, with adequate reliability values (Cronbach's Alpha between 0.71 and 0.87). In a subsequent validation study (Garzón Umerenkova et al., 2017a) using CFA and Rasch analysis, the dimensionality of the four subscales and the adjustment of the items to the model was adequate and the functioning of the measurement scale was confirmed. Reliability values of the measure above 0.95 were obtained for the four subscales; however, the reliability for persons and the construct validity could be improved by the inclusion of more items, both of greater and lesser difficulty.

To analyze the results of the study, in regard to SR, two measures were obtained: scores for each one of the four sub-dimensions and total SR level, sum of the four sub-dimensions.

#### Validated Spanish version of the flourishing scale (Diener et al., 2009)

The Spanish version of the “Flourishing Scale” (FS), previously validated for Colombian and Spanish populations (Pozo et al., 2016), was applied. The FS is intended to measure flourishing and consists of eight items, which have a five-point Likert scale that ranges from 1 (strongly disagree) to 5 (strongly agree) (See Annex I). The psychometric properties of the FS were satisfactory in a sample of students from Colombia and Spain. In the Colombian sample, the model obtained good fit indexes (CFI = 0.92, GFI = 0.92, NFI = 0.87). In the Spanish sample, the results were similar for the fit indexes (CFI = 0.95, GFI = 0.94, NFI = 0.91). The unidimensionality of the scale and the metric invariance in the evaluated samples was confirmed (RMSEA.062, CFI.940, and TLI.916). Cronbach's alpha for the Colombian sample was 0.88; and 0.85 for the Spanish sample.

#### Physical and psychosocial well-being inventory (health)

In order to obtain a self-report on the physical and psychological health of the participants, an *ad-hoc* questionnaire was created on which eight assertions were presented and are shown in Annex I. This inventory summarizes the definition of health by the World Health Organization (WHO): “Health is a state of complete physical, mental, and social well-being and not merely the absence of disease or infirmity.”^1^ It was considered to focus the questions on aspects related to the effects of the study, e.g., “*I feel anxious about my studies”* and avoiding redundant questions that may appear in the Flourishing Scale. Through these, an overall assessment of the general health of the participants was performed which examined feeding, sleep and recreation habits; besides anxiety, depression or stress that studies may be generating. A five-point Likert scale was used ranging from 1 (strongly disagree) to 5 (strongly agree). In the Colombian sample, the model obtained good fit indexes (CFI = 0.96, GFI = 0.94, NFI = 0.90; RMSEA = 0.072), with a Cronbach's alpha of 0.82.

#### Validated Spanish version of the procrastination assessment scale-students, PASS (Garzón and Gil, 2017)

The PASS test by Solomon and Rothblum (1984) consists of 44 items and is divided into two sections (See Annex I). The first is composed of 18 items that assess the *intensity* of procrastination. The second goes from item 19 through 44 and investigate the cognitive-behavioral reasons to procrastinate.

For the test validation in Colombia, a linguistic adjustment was made, and adequate reliability values were obtained (Cronbach's Alpha of 0.71–0.82); also, discriminant validity evidence was obtained for the procrastination frequency in function of time management and academic performance measures (Garzón and Gil, 2017). In another posterior validation study in a Colombian population (Garzón and de la Fuente, in review) for the second part of the test (item 19–44) a Confirmatory Factor Analysis (CFA) and a Rasch analysis were performed. Through CFA, adequate values for a three-factor model were obtained, which grouped the subjacent “reasons to procrastinate” (Rebellion, Anxiety, and Laziness). For these three factors and the items that constitute them, an adequate Rasch model fit was found, without any evidence of Differential Item Functioning (DIF) by gender or semester, but with evidence of discriminant validity on a self-regulation measure. Additionally, the results indicate that according to the underlying motive to procrastinate, there is a differential effect of impact on the self-regulation levels. The reasons to procrastinate were grouped in the three factors previously found for Colombian college population.

For the analysis of the results, in relation to procrastination, in the present study the following scores were obtained: (a) intensity level (procrastination frequency), as the sum of questions 1, 2, 4, 5, 7, 8, 10, 11, 13, 14, 16, and 17; (b) grouped scores of each one of the three reasons to procrastinate (questions 19 through 44); and (c) total procrastination, as the sum of the previous two.

#### Sociodemographic and academic performance questionnaire

Nine questions of sociodemographic variables were included as follows: gender, age, university, program, semester, daytime or nighttime studies, dedication to the studies (works and studies, studies full time), approximate cumulative grading average (from 1 to 5), and if the student plan his/her time (yes/no).

### Procedure

Testing was applied collectively in IT classrooms employing an online platform created for the study. The students participated voluntarily and were not encouraged. Taking into account the deontological and ethical psychology code in Colombia (Title 9, Research and Teaching, Article 50), informed consent from the participants was obtained and was previously approved by the *Research Commission of the Fundación Universitaria Konrad Lorenz*.

### Design and data analyses

An *ex post facto* design was used. A statistical analysis of bivariate Pearson correlation was carried out using SPSS (v. 23.0) for Windows. For *ag*e, two groups were formed*:* young (18–24) and older (25–41). AMOS (v. 23.0) for Windows was used for the structural validity analysis of each inventory and for constructing the structural model. To interpret the CFA and SEM model fit, we focused on the comparative fit index (CFI) and the root mean square error of approximation (RMSEA). CFI values equal to or more than 0.90 and 0.95 respectively were taken to indicate acceptable and close fit to the data (McDonald and Marsh, 1990). RMSEA values equal to or below 0.05 and 0.08 were taken to indicate close and acceptable levels of fit, respectively (Jöreskog and Sörbom, 1993). Keith (2006) proposed the following educational research benchmarks for *direct effects* in the form of beta coefficients: less than 0.05 is considered too small to be meaningful, above 0.05 is small but meaningful, above 0.10 is moderate, and above 0.25 is large. For *indirect effects*, we use Kenny's (2012) definition of an indirect effect as the product of two effects. Based on Keith's benchmarks above, for indirect effects, we considered the following: (a) correlations between 0.003 and 0.01 are small, (b) between 0.01 and 0.06 are moderate and (c) above 0.06 are large.

## Results

### Linear relation

#### Bivariate correlations

The results of bivariate correlations analysis showed a positive and significant association between *self-regulation* (SR), flourishing (FL) (0.390), and health (0.435) on both the physical (0.367) and psychological (0.361) components (See Table 2). An association, significant and negative, between self-regulation (SR) and procrastination (PRO) (−0.401), and between procrastination and performance (PER) (−0.162), was also evidenced. Notably, *flourishing* (FL) showed to be significantly associated with health (0.421) (both components, 0.458, and 0.421), but not with performance and only with the planning (PLAN) (0.167) conduct of it. *Procrastination* (PRO) was associated significantly and negatively with performance (PER) (−0.162), with dedication (DED), grade average (AVER), and planning (PLAN) (−0.247), but not with health (HEAL). *Health* (HEAL) was associated with performance (PERF) (0.129) and organization (ORG) (0.149). Lastly, *gender* was associated significantly and positively with flourishing (FL) (0.184) and performance (PERF) (0.173), and negatively with procrastination (PRO) (−0.178). *Age* appeared associated negatively with physical health (−0.170), and performance (−0.133), although with the latter being significant at *p* < 0.05.

**Table 2:** Bivariate correlations between variables.

	**SR**	**FL**	**HEAL**	**PHY**	**PSYC**	**PRO**	**PERF**	**DED**	**AV**	**PL**	**G**	**A**
SR												
FL	0.390											
HEAL	0.453	0.421										
PHI	0.367	0.458										
PSYC	0.361	0.231										
PRO	−0.401	−0.185	−0.280	−0.168	−0.275							
PERF	0.268		0.129^*^	0.142		−0.162						
DED	0.116^*^		0.206	0.285		−0.103^*^						
AVER	0.221					−0.127						
ORGA	0.421	0.167	0.149^**^	0.208		−0.247						
GEND		0.184				−0.178	0.173					
AGE				−0.170			−0.133^*^					

SR, Self-regulation; FL, Flourishing; PROCR, Procrastination (the sum of intensity and reasons to procrastinate); HEAL, Health; PER, Academic performance; F1–F8, Items of flourishing (see ANEX I); PHY HEALTH, Physical health; PSYC. HEALTH, Psychological health; DED, Dedication; AVER, Average; ORG, Organization; GENDER, Gender; AGE, Age; All the correlations are significant to p < 0.001;

** p < 0.01;

* *p < 0.05*.

### Multivariate relation pathway

The relation parameters of both models are set out below. The proposed *initial theoretical model* established that: (a) the latent variable self-regulation (SR) would significantly and positively predict the variables flourishing (FL) and health (HEL), (b) flourishing (FL) would positively predict health (HEAL), (c) health would predict performance (PERF), and (d) the latent variable self-regulation (SR) would significantly and negatively predict procrastination (PROC), and positively performance (PERF). Moreover, it was expected that the non-latent gender and age variables were predictors of all the latent variables studied (SR, FLOUR, HEALTH, PROC, and PERF).

For the *final empirical models*, two models were tested, obtaining more consistent results on the second which was taken as definitive. In *model* 1, the SR predictive relations in regard to performance (PERF); and health (HEAL) prediction in relation to PERF were included, but the adequacy indexes were not consistent. The results of pathway analysis (SEM) showed an acceptable *model 2* of the relationship between variables (see Table 3). In that model, the SR prediction in relation to PERF and the HEAL prediction in relation to PERF was eliminated. Also, only the predictions of gender and age that were significant were maintained (standardized regression; *p* < 0.001).

**Table 3:** Models of structural linear results of the variables.

	**Chi^2^**	**FG**	***p*<**	**NFI**	**RFI**	**IFI**	**TLI**	**CFI**	**HOELTER**	**RMSEA**
Model 1	588,606	(299–81): 218	0.000	0.804	0.752	0.871	0.832	0.876	0.169	0.078
Model 2	563,884	(299–79): 220	0.000	0.952	0.955	0.961	0.952	0.956	0.215	0.060

### Standardized direct effects

This predictive linear model establishes that latent variable *self-regulation* (SR) is a significant positive predictor of the latent variable *flourishing* (FL) (0.45), and positively predicted for the latent variable *well-being* (HEAL) (0.53). Complementarily, the latent variable *flourishing* (FL) is a significant predictor of the latent variable *well-being* (HEAL) (0.49). Also, *self-regulation* (SR) is a significant negatively predictor for the latent variable *procrastination* (PRO) (−0.64), and *procrastination* (PRO) is a negative predictor of *academic performance* (PERF) (−0.63).

Moreover, the non-latent variable *gender* appeared with a significant and positive predictive relationship among the latent *flourishing* (FL) variable (0.18), and a significant and negative predictive relation among the latent variable *well-being* (HEAL) (−0.19). Also, gender has a significant and negative predictive relationship among the latent variable *procrastination* (PRO) (−0.21). Complementarily, the non-latent variable *age* predicted a significant and negatively relationship among the latent variable *well-being* (HEAL) (−0.19), a negatively and significantly relationship with the latent variable *procrastination* (PRO) (−0.13), and a negatively and significantly relationship with the latent variable *academic performance* (PERF) (−0.13). All the variance of errors was significant (*p* < 0.001). Table 4 shows the *direct effects* of the variables inherent in the model.

**Table 4:** Standardized direct effects (default model).

	**SR**	**FL**	**HEALTH**	**PRO**	**PERF**	***Gender***	***Age***
GOALS	0.753						
TENACITY	0.647						
DECISION	0.721						
MISTAKES	0.484						
F1		0.733					
F2		0.720					
F3		0.752					
F4		0.620					
F5		0.703					
F6		0.775					
F7		0.713					
F8		0.619					
PHYS. HEALTH			0.634				
PSYC. HEALTH			0.451				
PROCRAST INTENSITY				0.416			
REASONS TO PROCRASTINATE							
REBELLION				0.591			
ANSIETY				0.597			
SLOTH				0.776			
DEDICATION					0.495		
AVERAGE					0.306		
ORGANIZATION					0.400		
FL	0.448					0.177	
HEALTH	0.533	0.495				−0.194	−0.189
PRO	−0.639					−0.211	−0.132
PERF				−0.630			−0.584

*SR, Self-regulation; FL, Flourishing; PRO, Procrastination; GENDER, Gender; AGE, Age; HEALTH, Health; PER, Academic performance; F1–F8, Items of flourishing (see ANEX I); PHY HEALTH, Physical health; PSYC. HEALTH, Psychological health. All the coefficients were significant at p < 0.001*.

### Standardized indirect effects

The model also contributed the existence of *multiple indirect* predictions among the variables. This predictive linear model establishes that the latent variable *self-regulation* (SR) is a positive predictor of all *flourishing* items, and a positive predictor of physical (0.479) and psychological (0.340) well-being, and is a positive significant predictor of the latent variable psychological *well-being* (HEAL) (0.222), a positive predictor of latent variable *performance* (PERF) (0.403). The latent variable *flourishing* was another indirect predictive which had a positive relationship with *physical* (0.314) and *psychological* (0.223) *health*. The latent variable *flourishing* (FL) was an indirect predictive and had a negative relation to all factors *of academic performance*. Also, *self-regulation* (SR) was a significant positive predictor (0.222) of *dedication*, (0.199) *average and (0.161) planning*, as factors of *academic performance* (PERF), and negative predictor to *intensity* (−0.266) and to all *reasons to procrastinate* items (PRO): rebellion (−0.378), anxiety (−0.382), laziness (−0.497).

Additionally, the non-latent variable *gender* negatively predicted the components of *well-being* (HEAL) and on the intensity and reasons to *procrastinate* (PRO), and positively predicted the elements of *academic performance* (PERF). Finally, the non-latent variable of *gender* also appeared with a negative indirect effect on *procrastination* (PRO) and the *academic performance* (PERF). See Table 5.

**Table 5:** Standardized indirect effects (default model).

	**SR**	**FL**	**HEALTH**	**PROCR**	**PERF**	***GENDER***	***AGE***
FL							
PRO							
GENDER							
AGE							
HEALTH	0.222						
PERF	0.403						
GOALS							
TENACITY							
DECISION							
MISTAKES							
F1	0.328						
F2	0.332						
F3	0.337						
F4	0.278						
F5	0.315						
F6	0.334						
F7	0.319						
F8	0.277						
PHY HEALTH	0.479	0.314		−0.067			
PSYC. HEALTH	0.340	0.223		−0.048			
PROCR. INTENSITY	−0.266			−0.088	−0.055		
REASONS TO PROCR							
REBELLION	−0.378			−0.125	−0.78		
ANSIETY	−0.382			−0.126	−0.079		
SLOTH	−0.497			−0.164	−0.102		
DEDICATION	0.123			−0.311	0.066	−0.248	
AVERAGE	0.199			−0.193	0.041	−0.153	
PLANIFICATION	0.161			−0.252	0.053	−0.201	

*SR, Self-regulation; FL, Flourishing; F1–F8, Items of flourishing (see ANEX I); HEALTH, Health; PHY HEALTH, Physical health; PSYC. HEALTH, Psychological health; PRO, Procrastination; PERF, Academic performance; GENDER, Gender; AGE, Age; All the coefficients were significant at p < 0.001*.

### Graphic representation of the structural model

**Figure 1:**
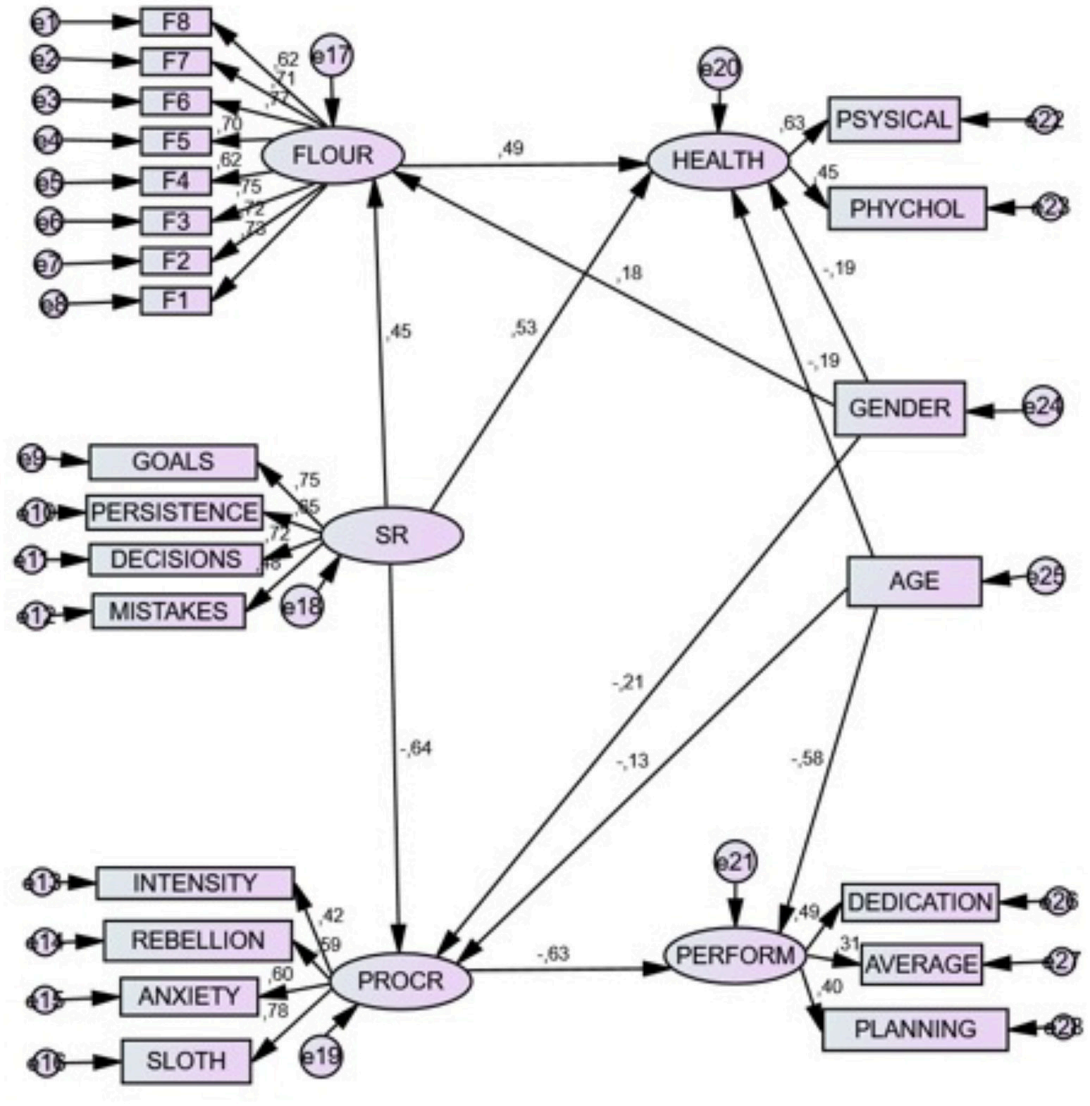
Structural relations between the studied variables: SR, Self-Regulation; FLOUR, Flourishing; HEALTH, Health; PROCR, Procrastination; PERFORM, Academic Performance. All the coefficients were significant at *p* < 0.01.

The final model is graphically represented in Figure 1.

## Discussion and conclusion

According to the aim of the study, concrete evidence of the bivariate correlation and structural prediction relation between the self-regulation levels and the study variables is provided. This study had, as a general objective, to establish the possible relations of linear association and structural prediction between: (a) self-regulation, flow and physical, and psychological health of students; (b) self-regulation and procrastination on academic performance, and (c) the role of gender and age. According to the methodology and the presented results, it is understood that determining, in an integrated and complementary way, the different psychological elements of individuals, leads to a broader and balanced view on the determinants of health (physical and psychological) and the academic performance of the students.

The proposed hypotheses at the beginning of the study were: (a) it is expected that self-regulation shows a positive linear relation of association and prediction in regard to flourishing and health (physical and psychological) of students; (b) it is expected to find a negative linear relation and prediction, between self-regulation, in regard to procrastination (frequency and reasons), and the performance of students; and (c) it is expected that gender and age have a relevant effect in the previously proposed relations.

The results have confirmed *hypothesis 1* showing that SR is positively associated, and is a positive predictor of flourishing and health, similarly to what previous investigations have confirmed (de Ridder and Kuijer, 2006; Mann et al., 2013). Likewise, given that the model indicates that flourishing predicts health, the results confirm that this construct goes beyond defining the emotional aspects of individuals, toward matters that have an impact on physical health and psychological well-being (Keyes, 2007), in this case in college students. This result is consistent with some of the previous evidence that already highlighted the important role that self-regulation has on psychological well-being (Howell, 2009; Vinothkumar et al., 2016) and in health (physical and psychological), especially in cases of chronic illnesses (de Ridder and Kuijer, 2006; Mann et al., 2013).

As established in *hypothesis 2*, procrastination was associated and predicted negatively by self-regulation. In that sense, procrastination can be adequately defined as a negative correlate of the lack of regulation, meaning, a self-regulation failure, as mentioned previously by other authors (Steel, 2007). These results are consistent, on one side, with the general character of procrastinating and low self-regulating students, who postpone their activities recurrently (Brownlow and Reasinger, 2000); and on the other side, with recent evidence that has shown that self-regulation level is associated with self-regulation learning (SRL) and with the academic performance of college students (de la Fuente et al., 2017b).

A confirmed negative association and prediction relation between procrastination and academic performance is shown and is consistent with previous studies that evidence the impact of procrastination on students' achievements (Garzón and Gil, 2017). On the other hand, although the structural relations do not confirm it, there is a small but significant inverse correlation between procrastination and physical and psychological health. The “reasons to procrastinate” impact on health could be modulated by the self-regulation levels, that in other studies with similar results, have been approached as “self-efficacy” (Kiamarsi and Abolghasemi, 2014). Likewise, although the structural model does not confirm it, there is a small but significant inverse correlation between flourishing and procrastination.

In regard to *hypothesis 3, gender* seems to have a substantial positive association and predictive value on *flourishing* (in favor of women), and negative association and predictive value on procrastination, and health. *Age* (in favor of the young) predicts positively health, procrastination, and academic performance. Differential results between men and women concerning intensity and type of academic procrastination have been previously reported in the literature (Özer et al., 2009, Garzón and de la Fuente, in review).

In general, the main objectives of the study were accomplished: (1) Offer a more sophisticated level of explanation and a superior predictive value of self-regulation on procrastination, health, and academic success of students. The results integrate and structure different factors to constitute predictive models that increase the capacity to comprehend on which factors the success and life quality of the students relies on. (2) These results allow the corroboration of the inherent conducts of self-regulatory and de-regulatory behaviors, as exposed in the SR vs. ERL Theory (de la Fuente, 2015, 2017) (See Table 1). In the first case, it has been evidenced that SR is associated positively and predicts the development of flourishing and health. In the second case, it has been proved that procrastination is negatively associated with self-regulation, which means that it can be considered as a proactive and negative behavior of self-impediment, as pointed out in the cited Theory.

### Limitations

Among the limitations of the study are the generalization of the results since only college students were considered, hence, for future studies a broader and more heterogeneous sample would be necessary for the model to be replicated. Also present is bias due to self-reporting measures. Concerning the improvement of the physical and psychological health, other measures could be included to establish if there are differential aspects in the model that are related to different health situations.

### Implications

According to the results, self-regulation is a personal and central construct in the prediction of the vital well-being (flourishing) and of health, as well as for the academic behavior of task delaying (procrastination) and the academic performance of college students. Therefore, the correct evaluation and training of self-regulation in educational contexts may benefit students' quality of life and improve their performance, from the perspective of education quality indicators that go beyond the concern of the students' grades. Complementarily, the results agree with the idea that sociodemographic variables like *gender* and *age*, together with psychological variables, may be employed by educational institutions to evaluate and formulate adequate intervention plans to different groups with varied “risk factors,” in light of this and other recent studies (Artuch-Garde et al., 2017).

### Future research directions

It is necessary to continue investigating the associations between the reasons to procrastinate and health and flourishing since the evidence points toward their existence.

However, considering the presented model, the role of SR on the relationships between these variables should be considered. To understand with more depth, the inverse relationship between self-regulation and reasons to procrastinate is necessary to explore the motivational base of procrastination and the different justifications employed by individuals to intentionally delay their tasks, in accordance to that presented by Kroese and de Ridder (2016). In future research, the deepening on procrastination and health measures has to be considered, as well as an approach to the individuals' reasons when procrastinating, in the context of the SR vs. ERL theory.

The differences found between men and women on the investigated factors in this study could have a motivational ground that affects procrastination as self-regulation and results in a better or worse academic performance. In a study by D'Lima et al. (2014) the authors found that women are more extrinsically motivated and mastery-oriented than their peer men, who were more performance oriented. Performance goal orientations are inversely associated with the average, while mastery orientation and intrinsic and extrinsic motivation are positively associated with the average. Self-regulation and the type of goal orientation have resulted predictive of academic achievement in other studies (Dekker et al., 2016), but the distinct differentiation by gender and age has not been approached. The exploration of the impact that these motivational aspects have by gender, and age group could be addressed in future research with greater depth, to establish their influence or moderation role on the relations presented initially on the proposed model.

## Author contributions

AG-U: Coordination of Project, Data collect, Final writing; JdlF: Final writing, Analysis of data, Data Collect; JA: Review research; PP, SF, and JF: Analysis of data; Review research.

### Conflict of interest statement

The authors declare that the research was conducted in the absence of any commercial or financial relationships that could be construed as a potential conflict of interest.

## Footnotes

^1^WHO. Terminology Information System [online glossary]. Available online at: http://www.who.int/healthsystems/hss_glossary/en/index5.html
