# Supplementary material for: A Linear Empirical Model of Self-Regulation on Flourishing, Health, Procrastination, and Achievement, Among University Students
Source: Front Psychol. 2018 Apr 13;9:536. doi: 10.3389/fpsyg.2018.00536 (PMC5909179; doi:10.3389/fpsyg.2018.00536)
Supplement: Supplementary file 1 [file DataSheet1.pdf]

ANNEX I.

FS (Flourishing Scale)

A series of statements are presented with which you can agree or disagree. Select the alternative that fits your opinion the best.

1. I lead a purposeful and meaningful life
2. My social relationships are supportive and rewarding
3. I am engaged and interested in my daily activities
4. I actively contribute to the happiness and well-being of others
5. I am competent and capable in the activities that are important to me
6. I am a good person and live a good life
7. I am optimistic about my future
8. People respect me

Procrastination Assessment Scale for Students (PASS)

Areas of Procrastination

For each of the following activities, please rate the degree to which you delay or procrastinate. Rate each item on an “a” to “e” scale according to how often you wait until the last minute to do the activity. Then indicate on an “a” to “e” scale the degree to which you feel procrastination on that task is a problem. Finally, indicate on an “a” to “e” scale the degree to which you would like to decrease your tendency to procrastinate on each task.

I. WRITING A TERM PAPER

1. To what degree do you procrastinate on this task?

Never	Almost Never	Sometimes	Nearly Always	Always
a	b	c	d	e

2. To what degree is procrastination on this task a problem for you?

Not At All	Almost Never	Sometimes	Nearly Always	Always
a	b	c	d	e

3. To what extent do you want to decrease your tendency to procrastinate on this task?

Do Not Want to Decrease		Somewhat		Definitely Want to Decrease
a	b	c	d	e

II. STUDYING FOR EXAMS

4. To what degree do you procrastinate on this task?

Never	Almost Never	Sometimes	Nearly Always	Always
a	b	c	d	e

5. To what degree is procrastination on this task a problem for you?

Not At All	Almost Never	Sometimes	Nearly Always	Always
a	b	c	d	e

6. To what extent do you want to decrease your tendency to procrastinate on this task?

Do Not Want to Decrease		Somewhat		Definitely Want to Decrease
a	b	c	d	e

III. KEEPING UP WITH WEEKLY READING ASSIGNMENTS

7. To what degree do you procrastinate on this task?

Never	Almost Never	Sometimes	Nearly Always	Always
a	b	c	d	e

8. To what degree is procrastination on this task a problem for you?

Not At All	Almost Never	Sometimes	Nearly Always	Always
a	b	c	d	e

9. To what extent do you want to decrease your tendency to procrastinate on this task?

Do Not Want to Decrease		Somewhat		Definitely Want to Decrease
a	b	c	d	e

IV. ACADEMIC ADMINISTRATIVE TASKS: FILLING OUT FORMS, REGISTERING FOR CLASSES, GETTING ID CARD

10. To what degree do you procrastinate on this task?

Never	Almost Never	Sometimes	Nearly Always	Always
a	b	c	d	e

11. To what degree is procrastination on this task a problem for you?

Not At All	Almost Never	Sometimes	Nearly Always	Always
a	b	c	d	e

12. To what extent do you want to decrease your tendency to procrastinate on this task?

Do Not Want to Decrease		Somewhat		Definitely Want to Decrease
a	b	c	d	e

V. ATTENDANCE TASKS: MEETING WITH YOUR ADVISOR, MAKING AN APPOINTMENT WITH A PROFESSOR

13. To what degree do you procrastinate on this task?

Never	Almost Never	Sometimes	Nearly Always	Always
a	b	c	d	e

14. To what degree is procrastination on this task a problem for you?

Not At All	Almost Never	Sometimes	Nearly Always	Always
a	b	c	d	e

15. To what extent do you want to decrease your tendency to procrastinate on this task?

Do Not Want to Decrease		Somewhat		Definitely Want to Decrease
a	b	c	d	e

VI. SCHOOL ACTIVITIES IN GENERAL

16. To what degree do you procrastinate on this task?

Never	Almost Never	Sometimes	Nearly Always	Always
a	b	c	d	e

17. To what degree is procrastination on this task a problem for you?

Not At All	Almost Never	Sometimes	Nearly Always	Always
a	b	c	d	e

18. To what extent do you want to decrease your tendency to procrastinate on this task?

Do Not Want to Decrease		Somewhat		Definitely Want to Decrease
a	b	c	d	e

Reasons for Procrastination

Think of the last time the following situation occurred. It's near the end of the semester. The term paper you were assigned at the beginning of the semester is due very soon. You have not begun work on this paper. There are reasons why you have been procrastinating on this task.

Rate each of the following reasons on a 5-point scale according to how much it reflects why you procrastinated at the time. Mark your answers on your answer sheet.

Use the scale:

Not At All Reflects Why I Procrastinated		Somewhat Reflects		Definitely Reflects Why I Procrastinated
a	b	c	d	e

19. You were concerned the professor wouldn't like your work.

20. You waited until a classmate did his or hers, so that he/she could give you some advice.

21. You had a hard time knowing what to include and what not to include in your paper.

22. You had too many other things to do.

23. There's some information you needed to ask the professor, but you felt uncomfortable approaching him/her.

24. You were worried you would get a bad grade.

25. You resented having to do things assigned by others.

26. You didn't think you knew enough to write the paper.

27. You really disliked writing term papers.

28. You felt overwhelmed by the task.

29. You had difficulty requesting information from other people.

30. You looked forward to the excitement of doing this task at the last minute.

31. You couldn't choose among all the topics.

32. You were concerned that if you did well, your classmates would resent you.

33. You didn't trust yourself to do a good job.

34. You didn't have enough energy to begin the task.

35. You felt it just takes too long to write a term paper.

36. You liked the challenge of waiting until the deadline.

37. You knew that your classmates hadn't started the paper either.

38. You resented people setting deadlines for you.

39. You were concerned you wouldn't meet your own expectations.

40. You were concerned that if you got a good grade, people would have higher expectations of you in the future.

41. You waited to see if the professor would give you some more information about the paper.

42. You set very high standards for yourself and you worried that you wouldn't be able to meet those standards.

43. You just felt too lazy to write a term paper.

44. Your friends were pressuring you to do other things.

Health Scale

I have good health

I sleep well

I have good feeding habits

I spend time with my family/friends

I combine adequately my study obligations with recreational activities

I feel anxious about my studies

I feel depressed about my studies

I feel stressed about my studies
